# SUMOylated Senataxin functions in genome stability, RNA degradation, and stress granule disassembly, and is linked with inherited ataxia and motor neuron disease

**DOI:** 10.1002/mgg3.1745

**Published:** 2021-07-14

**Authors:** Craig L. Bennett, Albert R. La Spada

**Affiliations:** ^1^ School of Life Sciences University of Lincoln Lincoln UK; ^2^ Departments of Pathology & Laboratory Medicine, Neurology, and Biological Chemistry, and UCI Institute for Neurotherapeutics University of California, Irvine Irvine California USA

**Keywords:** 53BP1, ALS4, AOA2, helicase, nucleolus, Senataxin, stress granule, SUMOylation

## Abstract

**Background:**

Senataxin (SETX) is a DNA/RNA helicase critical for neuron survival. SETX mutations underlie two inherited neurodegenerative diseases: Ataxia with Oculomotor Apraxia type 2 (AOA2) and Amyotrophic Lateral Sclerosis type 4 (ALS4).

**Methods:**

This review examines SETX key cellular processes and we hypothesize that SETX requires SUMO posttranslational modification to function properly.

**Results:**

SETX is localized to distinct foci during S‐phase of the cell cycle, and these foci represent sites of DNA polymerase/RNA polymerase II (RNAP) collision, as they co‐localize with DNA damage markers 53BP1 and H2AX. At such sites, SETX directs incomplete RNA transcripts to the nuclear exosome for degradation via interaction with exosome component 9 (Exosc9), a key component of the nuclear exosome. These processes require SETX SUMOylation. SETX was also recently localized within stress granules (SGs), and found to regulate SG disassembly, a process that similarly requires SUMOylation.

**Conclusion:**

SETX undergoes SUMO modification to function at S‐phase foci in cycling cells to facilitate RNA degradation. SETX may regulate similar processes in non‐dividing neurons at sites of RNAP II bidirectional self‐collision. Finally, SUMOylation of SETX appears to be required for SG disassembly. This SETX function may be crucial for neuron survival, as altered SG dynamics are linked to ALS disease pathogenesis. In addition, AOA2 point mutations have been shown to block SETX SUMOylation. Such mutations induce an ataxia phenotype indistinguishable from those with SETX null mutation, underscoring the importance of this modification.

## Graphical abstract

This mini‐review summarizes the most pertinent aspects of SETX biology that have recently been found to coalesce around its SUMO posttranslational modification. Senataxin (SETX) is a helicase that functions to maintain genomic stability, and SETX class‐specific mutations cause either a severe recessive ataxia (AOA2) or a dominant motor neuron disease (ALS4). RNA processing and binding proteins such as SETX remain of major focus in neurologic disease. Yet, the precise mechanism leading to pathology is elusive.

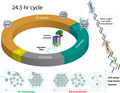

## 1 SETX GENE MUTATIONS IN NEURODEGENERATION AND INSIGHTS FROM STUDIES OF YEAST Sen1p

The senataxin (*SETX*) (OMIM: 608465) gene first gained attention in 2004 when a French group identified recessive mutations linked with a severe ataxia with oculo‐motor apraxia, AOA2 (Moreira et al., 2004). At the same time, we reported dominant SETX mutations linked to a rare form of juvenile Amyotrophic Lateral Sclerosis (ALS), known as ALS4 (Chen et al., 2004). The prefix “sen” was adopted, as homology was found between this new protein and the yeast helicase, Sen1p. The Sen was combined with “ataxia” to form Senataxin. Detailed clinical outlines for AOA2 (Le Ber et al., 2004) and ALS4 (Rabin et al., 1999) have been previously reported, and thus, they will not be discussed here.

From 2004 until now, the search to understand SETX function, and the disease mechanisms underlying AOA2 and ALS4 have been underway. Initially, we and others discovered that *SETX* was ubiquitously expressed (Chen et al., 2004; Moreira et al., 2004), highlighting the selective vulnerability of different neurons to either SETX loss‐of‐function (AOA2) or SETX gain‐of‐function (ALS4). Epitope‐tagged SETX constructs confirmed localization to the nucleoplasm (Chen et al., 2006; Yuce & West, 2013). A *Setx* gene knockout mouse (*Setx*‐KO) was made in 2013, but it lacked a neurological phenotype (Becherel et al., 2013). We produced ALS4 mouse models based upon the expression of wild type and R2136H isoforms driven by the mouse Prion Promoter transgenic promoter expression system, and by gene‐targeting to introduce the p.Leu389Ser (L389S) ALS4 mutation. These mice displayed mild motor phenotypes and TDP‐43 nuclear clearance pathology that is a hallmark feature of almost all human ALS patient cases (Bennett et al., 2018). However, like human patients, ALS4 mice have a normal life span. Disease phenotypes were limited to the neuro‐muscular axis as affected mice did not exhibit ataxia, seizures, or tremors. We did not observe increased motor neuron death, but soma area was reduced and the L389S knock in model also showed reduced axon diameter. The TDP‐43 nuclear clearance was present in ~10% of motor neurons. Thus, the ALS4 mice have potential utility for therapeutic trials, but the molecular basis of ALS4 disease pathogenesis remains elusive.

Prior to SETX‐specific studies, attention was focused on the yeast orthologue, Sen1p, and attempts were made to extrapolate function to SETX. However, the SETX protein sequence is longer at 2677 amino acids, and homology with Sen1p is limited to the 500 amino acid helicase domain only (Bennett & La Spada, 2015). Sen1p itself is well characterized and primarily functions in processing non‐coding RNAs (ncRNAs) (Ursic et al., 1995, 1997). The clearest demonstration of Sen1p function is in transcription termination (TT) of ncRNA’s (Rasmussen & Culbertson, 1998). For this, Sen1p utilizes the Nrd1–Nab3–Sen1p complex (Vasiljeva & Buratowski, 2006). But attributing a major role for SETX in TT is difficult as the key RNA‐binding protein Nab3 has no human homolog(Kuehner et al., 2011); and in vivo studies have ruled out a major role for SETX in this process (Banerjee et al., 2009). R‐loop resolution is suggested as another Sen1p defining function (Mischo et al., 2011). R‐loops naturally form when transcribed RNA strands temporarily hybridize with one DNA strand of an unwound duplex. Such R‐loop resolution has been demonstrated for SETX (Skourti‐Stathaki et al., 2011), but R‐loops that form in mammals tend to be significantly longer at ~1 kb or greater (Yu et al., 2003). Thus, with low RNA processivity, SETX would not be able to unwind very long structures in a genome‐wide, global sense (Han et al., 2017). Furthermore, R‐loop resolution in *Setx*‐KO mouse brain was found to be normal (Yeo et al., 2014). However, as R‐loop resolution has been demonstrated for SETX in general (see review Groh et al., 2017), R‐loop resolution may take place during certain SETX‐specific activities, as discussed below. But interestingly, studies using patient fibroblasts found that the p.Leu389Ser mutation led to reduced R‐Loop presence, suggesting that the ALS4 gain‐of‐function mechanism is in fact hyper helicase activity (Grunseich et al., 2018).

### 1.1 SETX functions at DNA Polymerase/RNA Polymerase II collision sites during S‐phase

**FIGURE 1:**
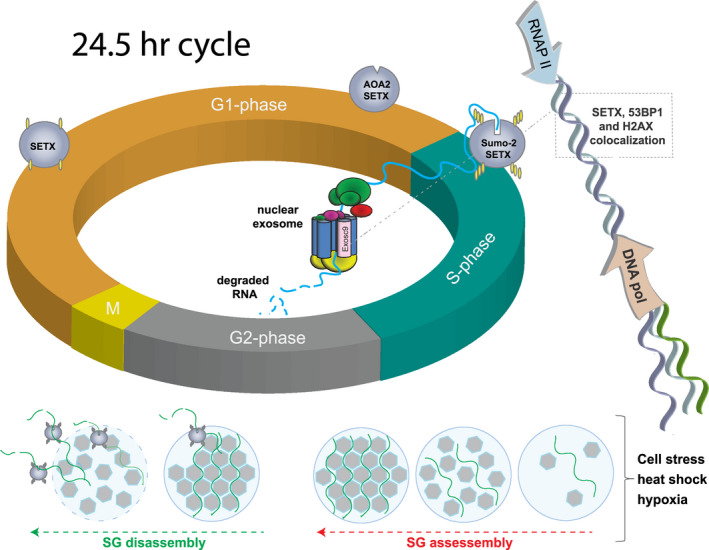
SUMOylation is critical for SETX function during S‐phase and for SETX‐regulated SG disassembly. The four major phases of the cell cycle are illustrated in the diagram. In early S‐phase, SETX shows a strong increase in SUMOylation (yellow rods). This is necessary for its interaction with Exosc9 of the nuclear exosome (pink cylinder in exosome complex), and SUMOylation is abolished by AOA2 point mutations (triangular indent; absent SUMO marks). Coordination of these events is essential, as SETX is thought to direct incomplete RNA transcripts to the exosome for degradation at sites of S‐phase foci (colocalizing with 53BP1 and H2AX). Such foci form when the DNA replisome collides with the RNAP II transcription machinery (blue and tan arrows). At the bottom of the diagram, SG assembly is indicated (light blue circles), with RNA‐binding proteins (gray hexagons), and mRNAs (green wavy lines) shown. During SG disassembly (green dashed arrow), SETX assists with this process by functionally interacting with specific mRNA clients

While the full list of SETX functions will require further detailed explanation, we are gaining a more comprehensive understanding of key SETX functions during S‐phase of the cell cycle. In all cycling cells, the DNA replication phase is known as the synthesis‐phase or S‐phase. But a less appreciated fact is that RNA transcription continues concurrently with DNA synthesis (Helmrich et al., 2011). During S‐phase, a number of independent research groups have shown that SETX forms nuclear foci indicative of replication stress at collision sites between the *DNA replisome* and the *RNA transcription machinery* (Bennett & La Spada, 2015; Skourti‐Stathaki et al., 2011; Yuce & West, 2013) (Figure 1). Formation of SETX foci is dynamic, as they are reduced by inhibiting RNA transcription (α‐amanitin), and increased by slowing the DNA replication fork (aphidicolin) (Yuce & West, 2013). These foci likely represent sites of DNA damage, with SETX colocalizing with 53BP1, a key DNA damage response protein, and with H2AX, a histone DNA damage marker (Yuce & West, 2013).

## 2 SETX REQUIRES SUMOYLATION TO BE DIRECTED TO S‐PHASE FOCI

To facilitate SETX localization and function, the ubiquitin‐like modifier SUMO is the strongest candidate. SUMO modification was first shown for SETX in 2006 (Hecker et al., 2006). In a heat shock proteomics analysis, SETX was found to be highly SUMOylated following this cell stressor (Bruderer et al., 2011; Golebiowski et al., 2009). We and others utilized the N‐terminus, protein interaction domain of SETX as bait in yeast two‐hybrid (Y2H) screens, and independently identified a number of SUMO pathway players, including: SUMO, SAE2, Ubc9, and the E3 SUMO‐protein ligases PIAS1 & TOPORS as SETX‐interacting proteins (Bennett et al., 2013; Richard et al., 2013). Finally, a highly sensitive SUMO‐2 proteomics analysis of the cell cycle was performed in 2014. To focus on this modification at all stages of the cell cycle, HeLa cells stably expressing Flag‐tagged SUMO‐2 were synchronized and were analyzed by mass spectrometry (Schimmel et al., 2014). Fully SUMO‐2 modified SETX increased in MW from ~306‐kDa to an apparent molecular mass of ~586‐kDa. Each SUMO‐2 monomer adds just 11‐kDa, so this represents a significant decoration. It should be noted that SUMO‐2/3 modification is typically chain links of ≥3 units. But most importantly, SETX was found to undergo a high level of SUMO‐2 modification dependent upon cell cycle phase (Figure 1); and specifically during early S‐phase, with other peaks at S/G2 and G2/M (Schimmel et al., 2014). Hence, SUMO‐2 modification likely promotes SETX foci to accumulate as this distinctive S‐phase feature. In addition, SETX assists in another critical process linked with SUMOylation, that of transcriptional silencing. During meiosis, this remodeling process occurs when genes located along un‐synapsed chromosomes are transcriptionally inactivated during prophase I. SETX loss causes a marked decrease in protein SUMOylation across the XY body during the pachytene stage of meiosis, and is itself SUMOylated (Yeo et al., 2015).

## 3 SETX SUMOYLATION IS REQUIRED FOR EXOSOME INTERACTION AND BLOCKED BY AOA2 MUTATIONS

With advancement of the DNA replication fork, a direct collision with RNA polymerase II (RNAP II) from the reverse direction will necessarily halt RNA elongation. At this point, SETX may utilize its conserved helicase function to unwind R‐loops, which form behind stalled RNAP II. Thereafter, SETX would direct incomplete RNA transcripts to the exosome for degradation. The exosome complex is the major eukaryotic 3′ → 5′ exonuclease, consisting of nine conserved core subunits that function in the accurate degradation of RNAs in the nucleus and cytoplasm (Sloan et al., 2012). Exosome Component 9, Exosc9, was highly represented in the two SETX Y2H screens outlined above (Bennett et al., 2013; Richard et al., 2013). It was noted that SETX and Exosc9 were unable to interact when using purified recombinant protein, suggesting that posttranslational modification is required for interaction (Richard et al., 2013). This group went on to characterize within the Exosc9 C‐terminus, a Sumo‐interacting motif (SIM) that is required for interaction with the SETX N‐terminus, the domain that is extensively SUMO‐2/3 SUMOylated. Three SETX AOA2 point mutations, p.Glu65Lys, p.Trp305Cys, and p.Pro413Leu (for protein domain location, see Figure 1 Bennett & La Spada, 2015) (Bennett & La Spada, 2015), were studied with targeted Y2H assays. Each of these mutations showed loss of interaction with Exosc9 and decreased SETX SUMOylation (Richard et al., 2013). Interestingly, two ALS4 gain‐of‐function mutations, p.Thr3Ile and p.Leu389Ser were also tested, and they showed no loss of interaction with Exosc9, nor loss of SUMOylation.

### 3.1 Over‐expression of tightly regulated SETX, induces S‐phase arrest

**FIGURE 2:**
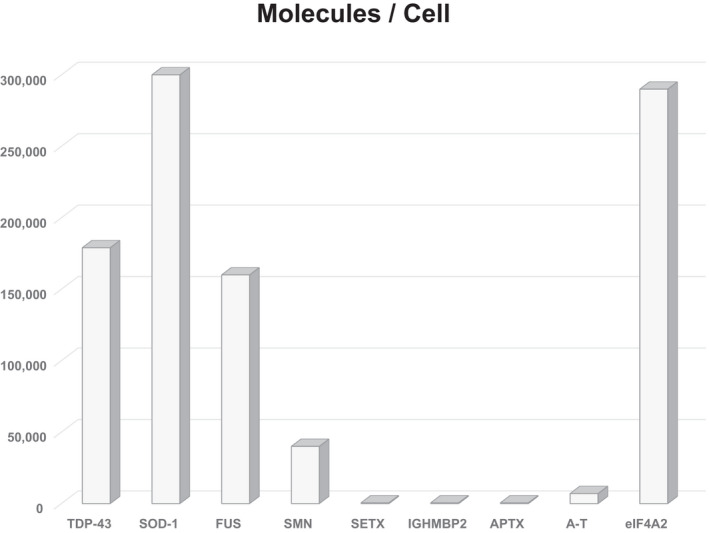
Total cell protein levels compared between ALS and Ataxia. Quantification of the approximate number of molecules per cell was compared for several inherited forms of ALS (TDP‐43, 179,000; SOD‐1, 310,000; FUS, 160,000; SMN, 40,000) and inherited ataxias (SETX, <500; IGHMBP2, <500; APTX, <500; A‐T, 7000); and the translation initiation factor, eIF4A2, 290,000. Relative protein levels were determined by mass spectrometry of ~10,000 proteins from the U2OS cell line. Adapted from Beck et al. (2011). Protein abbreviations: TDP‐43, TAR DNA‐binding protein 43; FUS, Fused in Sarcoma; SMN, Survival Motor Neuron; SETX, Senataxin; APTX, Aprataxin; A‐T, Ataxia Telangiectasia Mutated; and eIF4A2, eukaryotic translation initiation factor 4A2

Tight regulation of Sen1p was demonstrated when yeast transformation achieved high levels of *sen1* mRNA, yet little increase in Sen1p protein resulted (DeMarini et al., 1995). We observed similar effects for SETX in HeLa cells when large increases in GFP‐SETX mRNA failed to increase total SETX protein (unpublished data). Tight SETX regulation appears to also hold true in vivo. We generated WT‐ and R2136H‐SETX (p.Arg2136His) transgenic mice with strong transgene expression, yet immunoblot analysis of brain extracts revealed no increase in total SETX protein (Bennett et al., 2018). If Sen1p/SETX are tightly regulated, what are its cellular levels? Yeast Sen1p is typically as low as 125 molecules per cell (Kim et al., 1999): much less than its known interaction partners Nrd1p at ~19,000, and Nab3p at ~5800 (Ghaemmaghami et al., 2003). In a detailed proteomics study of ~10,000 proteins, SETX was found to be in the very low abundance category, at <500 molecules/cell (Beck et al., 2011). From this same study, it was found that proteins linked with ALS, and showing dominant inheritance were in the mid‐protein level range, while those typically linked with recessive ataxia were at the very lowest levels (Beck et al., 2011) (Figure 2).

Yet, despite tight SETX regulation, transient transfection can boost SETX levels on an individual cell basis. We found that by transient transfection of HEK293 cells, GFP‐SETX was localized to the nucleoplasm, as expected (Bennett et al., 2020). But surprisingly, the nucleolus marker fibrillarin was diffused within the nucleus, coincident with SETX transfection. Indeed, greater than 90% of GFP‐SETX positive cells showed diffuse fibrillarin staining, even with low GFP‐SETX fluorescence. This result was replicated when Flag‐SETX was used as the tagged construct. Furthermore, deletion of the SETX helicase GTP‐binding domain abolished this effect, suggesting the phenomenon is dependent upon an active helicase domain. Curiously, the SETX‐induced fibrillarin redistribution appeared typical of prophase induction (Hernandez‐Verdun, 2011), prompting us to test cell cycle progression with propidium iodide staining and flow cytometry. We found that GFP‐SETX positive cells were S‐phase arrested, without evidence of increased cell death (Bennett et al., 2020).

Taken together, we suggest the following events come into focus for SETX during S‐phase. SETX undergoes increased SUMOylation during early S‐phase; it interacts with the nuclear exosome in a SUMOylation‐dependent manner; and it directs RNA to the exosome for degradation at sites of DNA polymerase/RNAP II collision. SETX is tightly regulated at very low levels and if cellular levels are elevated above undefined thresholds, S‐phase cell cycle arrest is induced. Given that SETX foci also represent 53BP1‐positive DNA damage response sites, we speculate that elevated SETX levels trigger DNA damage checkpoints as part of S‐phase arrest.

## 4 SETX ROLE IN STRESS GRANULE DYNAMICS IS REGULATED BY SUMOYLATION

Stress granules (SGs) are found in the cytoplasm and consist of proteins and non‐translating mRNAs at high concentration. SGs may form during cell stress to temporarily pause translation initiation until conditions are more favorable. An unappreciated point is that SGs components may be cell type and stress‐specific (Marmor‐Kollet et al., 2020). However, it is known that many proteins linked with neurodegenerative disease, such as TDP‐43 and FUS, can alter SG dynamics (Taylor et al., 2016). Disease‐linked point mutations show clustering within the intrinsically disordered regions (IDRs) of these familial ALS disease proteins. Indeed, the IDRs of various RNA‐binding proteins are part of the viscosity‐based, maintenance of SG/cytoplasm barriers. To shed more light on SG disassembly, a recent study employed APEX2 proximity proteomics. Based on this sensitive method, SUMO ligases were discovered as novel SG disassembly engaged proteins (DEPs), and SUMOylation itself was found to regulate both SG formation and disassembly (Marmor‐Kollet et al., 2020). An important example is that SUMOylation of the translation initiation factor eIF4A2 was shown to contribute to SG formation (Jongjitwimol et al., 2016). In a new APEX2 proteomics study, 109 novel SG proteins were identified, including SETX (Marmor‐Kollet et al., 2020) (Figure 1). After SG induction with sodium arsenite treatment, SETX showed a 15‐fold and 10‐fold enrichment over the bait control with FMR1 and FXR1 SG baits, respectively, but not with G3BP1. SETX joined another group of 153 proteins identified in SGs at basal conditions (no sodium arsenite induction). Intriguingly, only 10 proteins including SETX were found with both FMR1 and FXR1 baits at basal conditions (Marmor‐Kollet et al., 2020). SETX was not part of a 30 pre‐stress seed protein list, but its presence at the earliest formation of FMR1 and FXR1 SG cores (1 of only 10 such proteins), suggests the importance of SETX for these structures.

The SG disassembly process was previously thought to rely on autophagy and ubiquitin pathways; however, new studies points to heat shock proteins and RNA helicases as key regulators. The DEPs were defined using FMR1‐APEX2 as bait after sodium arsenite washout for 30‐, 60‐, and 120‐min recovery. SETX was included as part of 22 DEPs tested for SG dynamics modification by siRNA knockdown and live‐imaging during disassembly. After 1 hr of recovery, concomitant with siRNA knockdown, 9 of the 22 DEPs resulted in a significant change in disassembly dynamics. SETX depletion resulted in SGs being ~40% larger versus siRNA control treatment (Marmor‐Kollet et al., 2020). Furthermore, DEPs were highly enriched for SUMOylation pathway members from E1 to E3, including: SAE1/2 (E1); UBC9 (E2); and the E3 ligase TOPORS, which are all known SETX interactors (Bennett et al., 2013; Richard et al., 2013). A key role for SUMOylation within SG dynamics was further evident when 2D08, an inhibitor of the SUMO E2 conjugating enzyme UBE2I (UBC9), was found to reduce SG disassembly in a dose‐dependent manner (Marmor‐Kollet et al., 2020). Finally, during SG disassembly, the expression of C9orf72 toxic arginine‐containing dipeptide repeats caused a depletion of DEP proteins including SETX and SUMO ligases (Marmor‐Kollet et al., 2020).

### 4.1 Conclusions

SETX is now recognized as a major contributor to genome stability and plays an important role during S‐phase of cycling cells. Its SUMOylation is necessary for Exosc9 interaction, a core component of the nuclear exosome. These two events appear necessary for SETX’s function at S‐phase foci where it facilitates exosome‐mediated degradation of stalled RNA transcription at DNA polymerase/RNAP II collision sites. Also, SETX co‐localizes with the DNA damage markers 53BP1 and H2AX at these foci. Because SETX is maintained at very low cellular levels, transient over‐expression may trigger cell cycle check points. But neurons do not replicate, and both AOA2 and ALS4 result primarily in neuron degeneration. AOA2 loss‐of‐function mutations appear to block both SUMOylation and Exosc9 interaction. Hence, unless RNAPII self‐collision is of major concern for neuronal pathogenesis, other functions of SUMO‐SETX must contribute to neurodegeneration. The obvious candidate for this dysfunction would be a role for SETX in the regulation of SG dynamics, a core pathological hallmark in various forms of neurodegeneration. Based upon recent work, we now know that SETX, when found in SGs in close proximity to FMR1 and FXR1, plays an important role in SG disassembly, likely by its helicase domain‐dependent function of unwinding certain SG RNAs. Identifying and characterizing the SG RNA clients of SETX will yield crucial insights into the pathobiology of neurodegenerative diseases characterized by SG dysregulation.

## CONFLICT OF INTEREST

The authors declare no conflict of interest.

## AUTHOR'S CONTRIBUTIONS

All authors contributed equally to this work.

## ETHICS STATEMENT

No human subjects or animals were used in the production of this review.

## Data Availability

Data sharing is not applicable to this article as no new data were created or analyzed in this study.
